# “Not Today”: Overcoming PrEP Hesitancy, Stigma, and Adherence Barriers With Lenacapavir

**DOI:** 10.31486/toj.25.5057

**Published:** 2025

**Authors:** Margaret Conrad, Meredith Clement

**Affiliations:** ^1^ Department of Internal Medicine, Emory School of Medicine, Atlanta, GA; ^2^ Section of Infectious Diseases, Louisiana State University Health Sciences Center, New Orleans, LA

*You ask your patient, Ms S, a 23-year-old female returning to your clinic for treatment of urogenital gonorrhea, if she is interested in learning more about pre-exposure prophylaxis (PrEP) today.*

*“The HIV medicine? Nah, I don’t need that.”*

*You explain that PrEP is an incredibly effective HIV prevention tool and that southeastern Louisiana has some of the highest rates of HIV in the country. Anyone who is sexually active here qualifies for HIV prevention with PrEP.*

*“Well, I’ll think about it. But* ***not today****.”*

Providers in Louisiana have frequent interactions with patients like Ms S: folks who are having condomless sex in a state with one of the highest HIV incidence rates in the country but who have what HIV prevention specialists colloquially refer to as *low risk perception*. In this scenario, patients believe they do not fit within the demographic groups they associate with HIV risk.

However, PrEP hesitancy is about more than risk perception. In the Deep South, HIV stigma plays a profound role in who we—both patients and providers—view as truly at risk for HIV. No one likes to see themselves as at risk for HIV because they have seen the consequences of living with this chronic infection. People living with HIV in Louisiana avoid disclosing their diagnosis to friends and family for fear of being ostracized—forced to eat on paper plates, kicked out of the house, or otherwise socially isolated. Stigma affects all aspects of HIV care; consequently, fear of testing and delayed diagnosis are seen all too often. As a consequence, 47% of the Louisiana population living with HIV carry a diagnosis of late-stage HIV/AIDS.^1^

Because of these challenges, convincing patients that oral PrEP is an appropriate choice for them can be difficult, despite the dramatic reductions in HIV seen with its use. The burden of daily pill-taking is not something many people are ready to take on; it is simply too much of a commitment when the perceived risk is low and HIV stigma is high.

Consider another common scenario:

*Mr J, a 26-year-old male, is newly diagnosed with HIV. He performed a home test after a partner told him he should get tested, and he came to the clinic for confirmatory testing. Previously, Mr J had been taking oral PrEP, but he self-discontinued 6 months ago. You don’t ask him about prior PrEP use, but he volunteers the information as he replays some recent life changes aloud. “I stopped taking it. Trying to keep up with school and work, I just couldn’t remember it every day. It was too much.”*

Patients like Mr J often deprioritize their own health in favor of other necessities, such as paying for food, housing, and other bills, or staying in school. Challenges to adherence include not just forgetting to take the pills but also the fear of being seen with the medication—here again, HIV stigma plays a role. In addition to competing priorities, other challenges to persistence in care include inadequate transportation, loss of insurance, and other access barriers that prevent patients from making it to routine PrEP appointments. In one study examining pharmacy-based data on oral PrEP prescriptions, only 54% of commercially insured PrEP users and only 30% of Medicaid PrEP users persisted in PrEP care at 1 year.^2^ For young people, especially those who are not already taking other daily medications, daily pill-taking just doesn’t fit into their lives filled with various other stressors.

Now imagine … a long-acting injectable with remarkable efficacy that can be administered only twice yearly. Nearly 100% of HIV infections were averted in 2 large clinical trials. Lenacapavir, a first-in-class capsid inhibitor, is a subcutaneous injection administered every 6 months that demonstrated superiority to daily oral PrEP in the PURPOSE1^3^ and PURPOSE2^4^ studies, both published in the *New England Journal of Medicine* in 2024.

In PURPOSE1, lenacapavir was studied among adolescent girls and young women (median age of 21 years) in South Africa and Uganda.^3^ The trial randomized 2,138 participants to lenacapavir and 1,070 participants to the oral PrEP medication tenofovir disoproxil fumarate (TDF/FTC, marketed as Truvada). The trial featured a counterfactual design, meaning that baseline HIV incidence was calculated for those screening into the study with the intention of comparing background HIV incidence to the HIV incidence for those on lenacapavir. For all participants testing positive for HIV at study entry, a recency assay was used to determine when HIV infection occurred, thus allowing an estimate of the background HIV incidence rate for study participants to be calculated. In PURPOSE1, the background HIV incidence estimate was very high, at 2.41 infections per 100 person-years. Remarkably, no HIV infections occurred in the lenacapavir group (0 infections per 100 person-years). Lenacapavir reduced incident HIV infections compared to the background HIV incidence (incidence rate ratio 0.00; 95% CI 0.00 to 0.04; *P*<0.001), which was the primary endpoint of the trial, and reduced incident HIV infections compared to TDF/FTC (incidence rate ratio 0.00; 95% CI 0.00 to 0.10; *P*<0.001), which was the secondary endpoint of the trial.

In PURPOSE2, lenacapavir was studied among men and gender-diverse persons who have sex with men (median age of 29 years) at 61 sites in 7 countries.^4^ Notably, New Orleans was a study site and recruited more than 20 participants into the trial. The trial randomized 2,183 participants to lenacapavir and 1,088 participants to TDF/FTC. The trial similarly used a counterfactual model, and the background HIV incidence rate in the screened population was 2.37 infections per 100 person-years. Two incident HIV infections occurred in the lenacapavir group (HIV incidence 0.10 per 100 person-years), and 9 incident HIV infections occurred in the TDF/FTC group (HIV incidence 0.93 per 100 person-years). Thus, the incidence of HIV infection in the lenacapavir group was significantly lower than either the background incidence (incidence rate ratio 0.04; 95% CI 0.01 to 0.18; *P*<0.001) or the incidence in the TDF/FTC group (incidence rate ratio 0.11; 95% CI 0.02 to 0.51; *P*=0.002).

The 2 infections in the lenacapavir group in the PURPOSE2 trial occurred following the first injection of lenacapavir (ie, not after delays in injection timing), with diagnosis at week 13 for the first participant and at week 26 for the second participant. Further pharmacokinetic analyses showed lenacapavir concentrations that were within expected range. Both participants were noted to have N74D capsid mutations at the time of diagnosis of HIV infection. While disconcerting to see HIV transmission occur outside of delayed injections, it is important to remember that HIV acquisition was a very rare occurrence. Notably, the 9 patients in the TDF/FTC group who were diagnosed with HIV had low or inconsistent adherence or had already discontinued TDF/FTC prior to diagnosis.^4^

The downsides of lenacapavir are few but worth discussion. In trials, injections were administered in the abdomen, and injection site reactions were consistently reported, including induration and erythema in 83.2% of participants.^4^ Injection-site nodules were also frequently seen and lasted an average of 183 days, meaning that for some participants, a new nodule developed just as the prior nodule was resolving. An issue with nodules is that they represent a visual sign of PrEP that may be objectionable to some patients. Currently, the open-label extension studies of the PURPOSE program are investigating the administration of injections in anatomic locations where the presence of nodules may be more subtle.

A twice-yearly injection may also result in less frequent screening for sexually transmitted infections (STIs). Prior studies have explored the potential tradeoff of delayed STI diagnoses with extending STI screening intervals from every 3 months to every 6 months. One study investigating bacterial STI prevention using doxycycline postexposure prophylaxis (doxy PEP) compared quarterly and biannual screening intervals.^5^ Among individuals in the doxy PEP arm, screening every 6 months instead of every 3 months led to 22.6 delayed diagnoses of asymptomatic STIs per 100 person-years. In patients not taking doxy PEP, screening every 6 months would have been associated with delayed detection of 47.7 asymptomatic infections per 100 person-years.^5^ Similarly, a modeling study evaluating STI screening frequency in the context of HIV PrEP found that quarterly screening reduced STI incidence by 50% compared to biannual screening.^6^ These findings highlight the need to balance improved HIV prevention adherence with the risk of delayed STI detection when extending screening intervals.

On June 18, 2025, the US Food and Drug Administration approved lenacapavir for the prevention of HIV.^7^ Gilead Sciences, Inc, the pharmaceutical company that makes lenacapavir, has pledged to implement an access strategy that prioritizes speed and availability for those who need the drug most. In October 2024, Gilead signed nonexclusive, royalty-free voluntary licensing agreements with 6 manufacturers to make and sell generic lenacapavir in 120 high-incidence, resource-limited countries.^8^ However, in the United States, the list price is $28,218 per year, higher than other PrEP medications: Descovy at $23,360 per year, Truvada at $22,265 per year, and cabotegravir at $24,755 per year.^9-11^ In sharp contrast, TDF/FTC (generic Truvada) can be priced as low as a few hundred dollars per year.^12^ Louisiana Medicaid is now approving lenacapavir, although the extent of coverage by some insurers is still uncertain. The United States Preventive Services Task Force (USPSTF) has given HIV prevention with PrEP a grade A recommendation, and the Affordable Care Act requires health plans to cover all USPSTF-recommended preventive services; however, prior approval is sometimes still required by some plans.

The introduction of a twice-yearly PrEP injection represents a transformative innovation with the potential to become the new standard of care. Many patients face substantial barriers to traditional oral PrEP, including HIV-related stigma, difficulty adhering to a daily pill regimen, limited access to health care, and a general aversion to daily medication. Lenacapavir offers a discreet, low-burden alternative for eligible patients. As demonstrated in our young male patient whose life challenges precluded use of a daily medication, providers routinely encounter individuals who acquire HIV despite having previously been prescribed oral PrEP. Lenacapavir will provide a novel and effective option that can overcome barriers to adherence and perhaps to retention in care. Just as highly active antiretroviral therapy marked a turning point in HIV treatment history, lenacapavir stands poised to do the same in HIV prevention.

“Not today” is what providers too often hear when discussing HIV PrEP with patients. Lenacapavir offers a powerful opportunity to reimagine HIV prevention—with a medication that can help patients overcome hesitancy, stigma, and adherence barriers—**today**.
